# Parenclitic and Synolytic Networks Revisited

**DOI:** 10.3389/fgene.2021.733783

**Published:** 2021-10-20

**Authors:** Tatiana Nazarenko, Harry J. Whitwell, Oleg Blyuss, Alexey Zaikin

**Affiliations:** ^1^ Department of Mathematics and Institute for Women’s Health, University College London, London, United Kingdom; ^2^ National Phenome Centre and Imperial Clinical Phenotyping Centre, Department of Metabolism, Digestion and Reproduction, Imperial College London, Hammersmith Campus, London, United Kingdom; ^3^ Section of Bioanalytical Chemistry, Division of Systems Medicine, Department of Metabolism, Digestion, Imperial College London, South Kensington Campus, London, United Kingdom; ^4^ Lobachevsky State University of Nizhny Novgorod, Nizhny Novgorod, Russia; ^5^ World-Class Research Center “Digital Biodesign and Personalized Healthcare”, Sechenov First Moscow State Medical University, Moscow, Russia; ^6^ School of Physics, Astronomy and Mathematics, University of Hertfordshire, Harfield, United Kingdom; ^7^ Department of Pediatrics and Pediatric Infectious Diseases, Institute of Child’s Health, Sechenov First Moscow State Medical University (Sechenov University), Moscow, Russia

**Keywords:** networks, graphs, parenclitic, synolytic, complexity

## Abstract

Parenclitic networks provide a powerful and relatively new way to coerce multidimensional data into a graph form, enabling the application of graph theory to evaluate features. Different algorithms have been published for constructing parenclitic networks, leading to the question—which algorithm should be chosen? Initially, it was suggested to calculate the weight of an edge between two nodes of the network as a deviation from a linear regression, calculated for a dependence of one of these features on the other. This method works well, but not when features do not have a linear relationship. To overcome this, it was suggested to calculate edge weights as the distance from the area of most probable values by using a kernel density estimation. In these two approaches only one class (typically controls or healthy population) is used to construct a model. To take account of a second class, we have introduced synolytic networks, using a boundary between two classes on the feature-feature plane to estimate the weight of the edge between these features. Common to all these approaches is that topological indices can be used to evaluate the structure represented by the graphs. To compare these network approaches alongside more traditional machine-learning algorithms, we performed a substantial analysis using both synthetic data with *a priori* known structure and publicly available datasets used for the benchmarking of ML-algorithms. Such a comparison has shown that the main advantage of parenclitic and synolytic networks is their resistance to over-fitting (occurring when the number of features is greater than the number of subjects) compared to other ML approaches. Secondly, the capability to visualise data in a structured form, even when this structure is not *a priori* available allows for visual inspection and the application of well-established graph theory to their interpretation/application, eliminating the “black-box” nature of other ML approaches.

## 1 Introduction

In the era of increasing large and complex (multi-modal) datasets (biological, climatic, medical, etc.), network approaches are becoming very popular. Indeed, representation of complex data in the form of a network, i.e. a graph with nodes and edges, is a powerful tool to visualise data structure, clusters and communities, and all other interdependencies. Graph theory, well established by mathematicians, provides many topological indices to describe possible features of a network. This is especially valuable for complex biological systems, when often some non-specific change can be compensated by changes in other regions of a connected network. By evaluating topological features, the transition between two states such as health or disease be detected. A clear difficulty in this analysis is how to represent the data in the form of a network if links between nodes-features are unknown? Several approaches have been recently suggested and applied to different cases of data analysis.

One approach is correlation graphs, where edge weights are proportional either to the correlation coefficient between the corresponding vectors of features [for a discussion, see Gorban et al. (2021)] or to the correlation between nodes, if each node has some internal structure, e.g. in the case of intra-gene methylation profiles (e.g., see Bartlett and Zaikin, 2016; Bartlett et al., 2014). Recently, a new network approach has gained popularity, first described by Zanin and Boccaletti (2011) and called a *parenclitic* network representation, from the Greek term for “deviation”. The main idea of this approach is to establish links between parameters (nodes) without any *a-priori* knowledge of their interactions (Zanin and Boccaletti, 2011) by using residual distances from linear regression models constructed between every pair of parameters as edge-weights. Networks constructed from this linear regression parenclitic approach (LRPA) have been successfully applied to different biological problems. For example, the detection of disease-related genes and metabolites (Zanin and Boccaletti (2011); Zanin et al., 2012; Zanin et al., 2013a; Zanin et al., 2013b; Zanin et al., 2016), brain research (Papo et al., 2014), and to identify signatures of cancer development from human DNA methylation data (Karsakov et al., 2017).

However, for many biological data structures, there is no linear dependence between features, and thus defining a graph in such as way makes interpretation impossible. To overcome this, alternative approaches have been developed. First, it was suggested to use 2-dimensional kernel density estimation (2DKDE) to model the control distribution (KDE Parenclitic approach, KDEPA). This methodology was successfully applied to the problem of diagnosing patients with Ovarian Cancer. (Whitwell et al. 2018).

The advantage of KDEPA over LRPA is that pairs of features do not necessarily have to be correlated, or even grouped into a single cloud. At the same time, KDEPA also has some drawbacks: it is difficult to correctly extend the density distribution beyond what is defined by the underlying data (unlike linear regression which can be extrapolated simply) and, similarly to LRPA, the selection of a threshold (common for all edges) or thresholds (different for each edge) when converting to a binary network for class separation.

As a further development, in Krivonosov et al. (2020) we have introduced a variation of parenclitic networks, that can be called *synolytic* from the Greek word for “ensemble”. In some sense, synolytic networks is a graph representation of the simultaneous action of multiple classifier ensembles. We demonstrated previously that any machine learning methods [e.g. support vector machine (SVM)] can be used as the core of the parenclitic approach (a function that describes the separation of controls and cases groups in the plane of two features). We proposed a software implementation with a choice of any kernel and demonstrated its ability to detect the DNA methylation signature of Down Syndrome disease. Moreover, we showed that the characteristics of the constructed networks help to interpret the obtained signatures in relation to aging in individuals from non-Downs Syndrome and Down Syndrome populations. A further development came from not binary networks, but weighted networks, and this method was successfully applied to prediction of survival for severely ill Covid-19 patients (Demichev et al., 2021), and for prediction of prostate cancer progression in patients on active surveillance (Sushentsev et al., 2021). We used SVM as the core, and the probability of belonging to a group of cases as the weights of the edges.

The weighted synolytic network approach (wSA) automatically solves the inherent drawback of KDEPA by normalizing the distance measure in terms of probabilities. Herein, we show that the synolytic approach is comparable, and sometimes better than other machine learning (ML) models. One advantage is in the visualization of results, which allows one to visually identify key features (see examples on Figure 2A, producing greater transparency to “black-box” ML algorithms. They offer the opportunity for applying more sophisticated network analysis concepts to study the resulting networks and allow the analysis of networks over time, leading to future ideas of parenclitic-longitudinal data analysis.

In this paper, we compare weighted (w) parenclitic (wLRPA, wKDEPA) and synolytic (wSA) parenclitic models with each other and with other ML methods for solving binary classification problems.

To compare the approaches, we used

### 1.1 Synthetic Data

Models of N-dimensional spheres of radius 1, where points of the inner sphere of radius 0.5 are denoted by Controls (that is, with class 0), and points with a radius from 0.5 to 1 are denoted by Cases (that is, with 1 class). This model structure was chosen to generate synthetic data to fairly compare different machine learning algorithms within a defined and understandable dataset. To this end, the data is easily visualized in dimensions 2 (a regular circle on a plane) and 3 (a sphere in three-dimensional space), and further they can be easily expanded to any data dimensions in accordance with an understandable principle. In such a model, the radius is a characteristic, implicitly sewn into the full vector of each sample, and which is a measure of the distance of a point from the centre of the sphere, and it is *a priori* known how far each point is from the spatial multidimensional boundary of the class division.

In addition to “ideal spheres”, we also consider “noisy spheres”—spheres with the addition of 50 random variables and “broken spheres”—N-dimensional spheres from which N/2 parameters were replaced with random variables (to study how the models work on data that do not contain the full set of parameters responsible for the difference between the two classes). For each approach separately, we discuss how to choose the best characteristics (providing better quality separation of classes) and then check how these conclusions are reproduced on real data sets.

Studying the characteristics of networks on these data, we initially evaluate how the characteristics of these networks correlate with their radii (that is, how well the network approach reads this implicit characteristic). The higher the correlation, the greater the class separation will be. Confirmation that the characteristics of networks are correlated with radii is an important validation of the correctness of the transformation of raw data to the networks.

We compare parenclitic approaches with other ML models by comparing the results of applying ML models to matrices of raw (initial) data and matrices of strengths (degrees) of vertices derived from the graph-structured determined by the parenclitic model. This showed that models using parenclitic vertex strengths are superior to models on raw data in situations where the sample size is significantly lower than the dimension of the data.

### 1.2 Real Data

Realizing that the model of synthetic data we have chosen has a very simple structure and the results on it may not be reproduced on real data, we collected a collection of 16 datasets of different dimensions of features (which were randomly selected from the repository of existing datasets), in which the size of the minimum class was >40 samples. We found that wLRPA and wKDEPA approaches did not perform as well in real datasets with a large dimension (in comparison to sample size), whereas the wSA synolytic approach did.

The results obtained in this work create a reliable basis for the application of synolytic approaches to real data. We especially emphasize here the use of such approaches to clinical omics data, where, as a rule, the sample size is typically small in relation to the number of features, which nevertheless can contain a rich source of diagnostic information. We show the advantage of using synolytic approaches to solve classification problems, but we note that the advantage also lies in the fact that this approach allows the visualisation of each patient in the form of a network and opens up additional possibilities for the study of such states using graph theory and network analysis.

## 2 Materials and Methods

### 2.1 Generation of Different Types of Synthetic Data

Synethetic data was generated using a sphere-model, For all modelling, we considered all possible combinations of *Sphere Dimensions*: (2, 3, 10, 30, 60, 90, 120, 150); number of *Case TRAIN samples*: (15, 65, 115, 165, 215, 265) and number of *Controls TRAIN samples*: (15, 65, 115, 165, 215, 265). Numbers of *Case TEST samples* and *Controls TEST samples* were calculated as 25% of corresponding *TRAIN* numbers.

#### 2.1.1 Ideal Spheres Model

Common model: area bounded by a N-dimension sphere of radius 1 (i.e. each *i* sample is represented by vector $X_{i}^{1},X_{i}^{2},\ldots ,X_{i}^{N}$ , where $R=\sqrt{{X_{i}^{1}}^{2}+{X_{i}^{2}}^{2}+\cdots +{X_{i}^{N}}^{2}}\leq 1$ ). We define *Controls* as points with radius 0.01 ≤ *R* ≤ 0.5 and *Cases* as points with radius 0.5 ≤ *R* ≤ 1.

#### 2.1.2 Noisy Spheres Model

Each N-dimension “Ideal Sphere” was expanded to 50 ”noise” variables (i.e. each sample is represented by vector $X_{i}^{1},X_{i}^{2},\ldots ,X_{i}^{N},V_{i}^{1},V_{i}^{2},\ldots ,V_{i}^{50}$ , where *V* ^*j*^ - vector of random values from uniform distribution in (−1, 1))

#### 2.1.3 Broken Spheres Model

Each N-dimension “Ideal Sphere” was “broken”: we only kept half of the variables from there and the other half was changed to random values (i.e. each sample is represented by vector $X_{i}^{1},X_{i}^{2},\ldots ,X_{i}^{\frac{N}{2}},V_{i}^{1},V_{i}^{2},\ldots ,V_{i}^{\frac{N}{2}}$ , where *V* ^*j*^—vector of random values from uniform distribution in (−1, 1))

All generated data are publicly available and described in the Supplementary Materials.

### 2.2 Real Data List

Real datasets were obtained from https://archive.ics.uci.edu and are presented in Table1.

**TABLE 1:** Real datasets description.

N	Dataset	Number of				Area
		Features	Samples	Cases	Controls	
1	Banknote Authentication (2013)	4	1,372	610	762	Computer
2	Blood Transfusion Service Center (2008)	4	748	178	570	Business
3	Vertebral Column (2011)	6	310	210	100	Medicine
4	Breast Cancer Wisconsin (Diagnostic) (1995)	10	699	241	458	Medicine
5	Indian Liver Patient Dataset (ILPD) (2012)	10	583	167	416	Medicine
6	Planning Relax (2012)	12	182	52	130	Computer
7	Climate Model Simulation Crashes (2013)	18	540	494	46	Physical
8	Diabetic Retinopathy Debrecen (2014)	19	1,151	611	540	Medicine
9	SPECTF Heart (2001)	22	267	212	55	Medicine
10	Ionosphere (1989)	33	351	126	225	Physical
11	QSAR Biodegradation (2013)	41	1,055	356	699	Chemical
12	SPECTF Heart (2001)	44	267	212	55	Medicine
13	Connectionist Bench (Sonar, Mines vs. Rocks) (1988)	60	208	97	111	Physical
14	Mice Protein Expression (2015)	77	1,080	510	570	Medicine
15	Urban Land Cover (2014)	147	675	122	553	Physical
16	Arrhythmia Data Set (1998)	260	452	245	207	Medicine

The same pre-processing was performed for all datasets:

• All missing values were replaced with the mean of the feature column;
• The features, with standard deviation equal to 0 have been removed;
• If the data was a non-binary classification problem, then the response vector was transformed into a binary one (by highlighting one of the classes): Cortex: “Ts65Dnc”—cases, other controls; Ionosphere: “g”—controls, other cases; QSAR: “RB”—cases, other controls; SONAR: ‘R’ - cases, other controls; URBAN: “building”—cases, other controls; Vertebral- 2c: “AB”—cases, other controls);
• If the data contained a preliminary division into TRAIN and TEST subsets (SPECT, SPECTF and URBAN), then they were collected into a single dataset and TRAIN/TEST labels were disregarded
• For each dataset, we repeatedly (20 times) produced random subsets of 80 samples with equal numbers of Cases and Controls, and then Test and Train labels were assigned equally in each class (that is, each subset consisted of 20 TRAIN Case samples, 20 TRAIN Controls samples, 20 TEST Case samples and 20 TEST Controls samples). Thus, a total of 320 (16 original datasets * 20 subsets) datasets of different feature dimensions, but the same sample size, were obtained.

All collection of real data and selected subsets of them are publicly available and described in the Supplementary Materials.

### 2.3 Parenclitic Approaches

In this analysis we have used three different Parenclitic networks architecture:

#### 2.3.1 wLRPA

**FIGURE 1:**
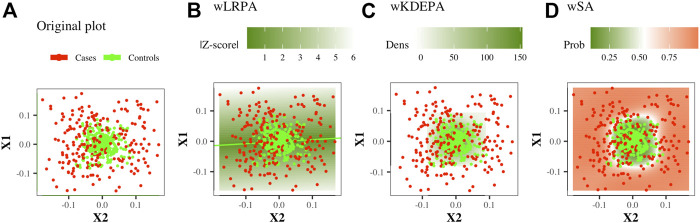
Illustration of the construction of parenclitic models on a pair of features (*X* ^1^, *X* ^2^) from the *Ideal ball model* with 165/265 Case/Control TRAIN points and 50/80 Case/Control TEST points. Case/controls are red/green points respectively.

This is a network where only the control group was considered as the basis for determining the normal state on the plane of two features: based on the control group, linear regression were built for every pair of features. The deviation of the control points from it was calculated and the distribution of such deviations was constructed (Figure 1B). In previous studies, for each new sample, the edge weight was first determined as the absolute value of z-score, and then binarized (if |*Z*−*score*| < 3, then the edge is present in the sample network, otherwise there is no edge). In this work, we will consider weighted networks (that is, the specified binarization will not be carried out, an edge for any sample will always exist and the edge weight will always be equal to |*Z*−*score*|).

#### 2.3.2 wKDEPA

This is a network in which again only the control group was considered as the basis for determining the normal state on the plane of two features. For the control group, the 2-dimension kernel density estimation was built for each pair of features (Figure 1C), then a function was calculated that converts the density values into an analogue distance, so that the points located in the area of the highest density have the minimum weight. The distance outside the grid was continued (for more details, see Whitwell et al., 2018). For non-weighted networks, for each new sample, the edge weight was first determined as the normalised volume of the density distribution above the point, and then converted to binary form (if the volume is greater than a threshold (which was iteratively selected, so that the characteristics of the resulting networks optimally separate the Case and Control groups), then the edge is present in the sample network, otherwise there is no edge). In this work, we will consider weighted networks (that is, the specified binarization will not be carried out, an edge for any sample will always exist and the edge weight will always be equal to a function of density).

#### 2.3.3 wSA

This is a network in which both groups participate in definition of normal and abnormal states. On the plane of any two features, a radial SVM is used to define the best boundary separating the classes (Figure 1D). Automatically, each point in such a model gets a value for the probability of belonging to each class. For each new sample, the edge weight is determined as the probability of belonging to a group of cases.

#### 2.3.4 Networks Characteristics

All characteristics were calculated with using *igraph package* (R).

The values for some characteristics were equal to *NA*, + *Inf*, − *Inf*, in these instances, the values were replaced by 0. Such substitutions could theoretically lead to the loss of differences between classes for some characteristics, although they did not affect the analysis associated with the Strengths of the vertices (these values are always are finite, since the Strength of the vertex is equal to the sum of the weights of the edges included in it).

For each dataset (model spheres and sets of real data), we built model-networks (i.e. built LM, KDE or SVM models in a plane of every pair of features) on the TRAIN folds. Networks were then constructed for each individual (TRAIN and TEST) sample in the dataset and for each of them we calculated:

• Descriptive statistics (*zeros, min, max, mean, standard deviation (sd), coefficient of variation (coefvar) = sd/mean*) of the main network characteristics closeness, betweenness, edge betweenness, page rank, eigen centrality, authority score, strength, edge weights;
• The full vector of strengths (degrees) of the vertices.

We use descriptive statistics of the main network characteristics to demonstrate their correlation with radii on synthetic data (and, as a consequence, the quality of class division into them). We use matrices of full vectors of strengths of vertices for samples in each dataset to compare the results of ML models on them and on the initial raw data.

### 2.4 ML Models for Comparison with Parenclitic Models

Parenclitic approaches were compared with 3 ML models (*xgbTree*, *nnet*, *glmnet*) from the Caret package in R). We chose these since the principles of their training are based on different static principles and they all produce a selection of features. We trained models using the *train* function within the Caret package, using scaling and centering for data pre-processing, with the selection of hyperparameters set at default and using 5-fold cross-validation.

All networks and their characacteristics from real data and selected subsets of them are publicly available and described in the Supplementary Materials.

### 2.5 Performance Estimation

The performance of ML models was assessed using area under the receiver operator characteristic curve (AUC).

For each dataset (synthetic or real, matrices of raw data or matrices of vertex degrees), we built 3 ML models on TRAIN folds and applied these models to TEST folds. For each result on TEST folds we calculated AUC with “direction” (i.e. *controls* < *cases* or *controls* > *cases*) received on AUC for TRAIN folds. Taking into account the direction along the TRAIN folds results in TEST-fold AUC values <0.5 in some instances.

To calculate the performance of class separation on each characteristic on synthetic data (Figure 3A), we use a simple *glm* model on each characteristic separately (obtaining AUC for TEST as described above).

All results (on synthetic or real, on matrices of raw data or matrices of vertex degrees, on separate characteristics) are publicly available and described in the Supplementary Materials.

To highlight the significance of the difference in the results obtained by ML models on raw data and on the degrees of vertices, a two-sided paired Wilcoxon signed-rank test was applyed to AUC values to calculate a *p*-value.

## 3 Results

### 3.1 Comparison of Approaches on Model Datasets

#### 3.1.1 Parenclitic

Since the data models were generated in such a way that the characteristic that distinguishes the classes (the radius of the spherical model) is always known, we first investigated how topological characteristics of the networks correlate with these values.

For each point in the sphere-models, the distance from the point to the centre of the *N*-dimensional data (radius) is calculated (“ideal sphere”) (see Section 2.1 for further details). To mimic non-perfect data, “noisy spheres” are also generated, in which 50 random features are added to each sample and “broken spheres” in which *N*/2 parameters are replaced with random variables. The radius for each point in “noisy spheres” and “broken spheres” are not recalculated, and thus the radius value (calculated for each point in the “ideal spheres” data) becomes a less accurate representation of the points position in the data structure.

**FIGURE 2:**
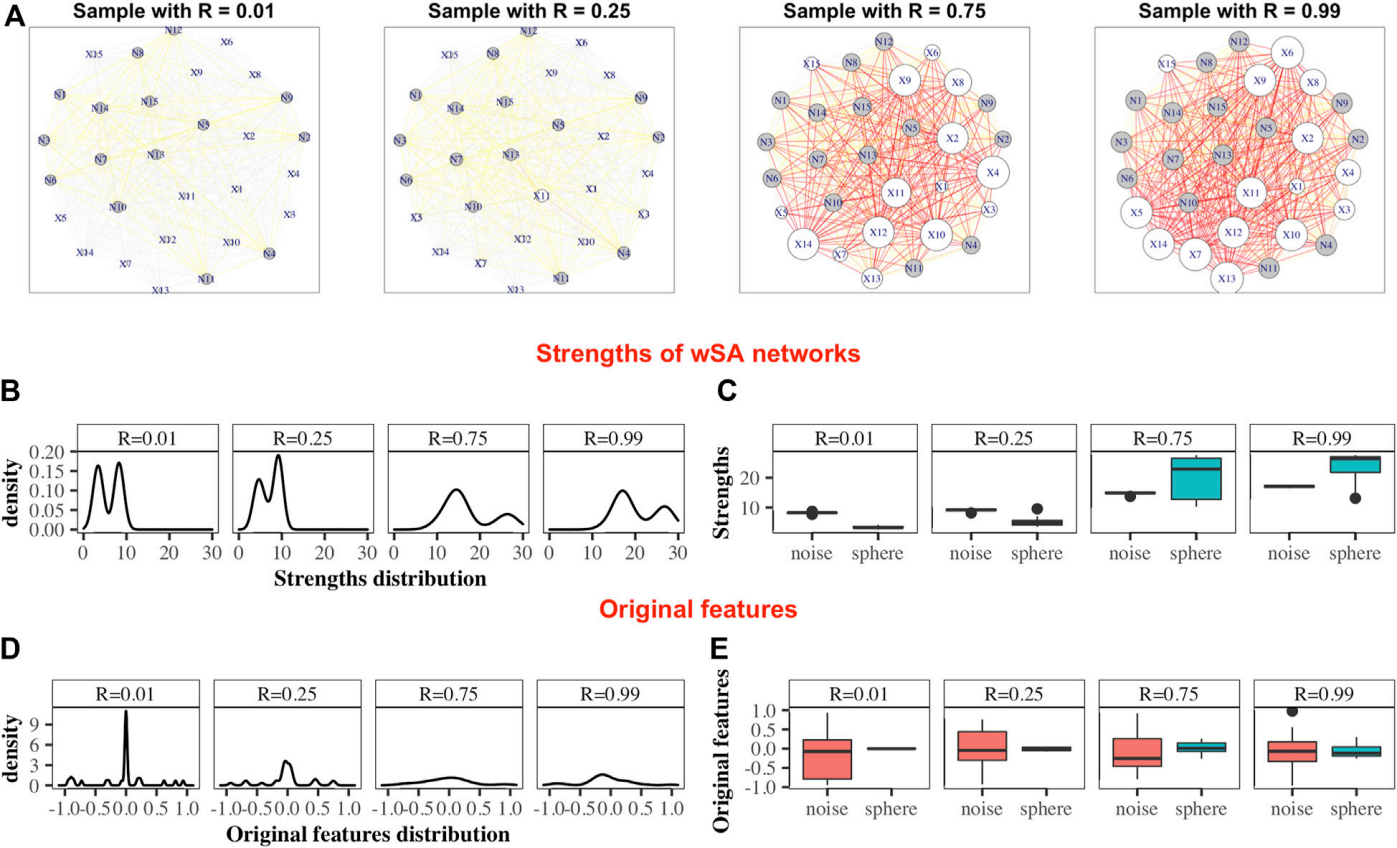
Demonstration of the advantages of the wSA approach: visualization of parameters in the form of networks. Examples wSA networks for samples with *R* = (0.01, 0.25, 0.75, 0.99) from a *broken sphere model* with 30-dimensions. There were 115/165 Case/Control TRAIN points and 34/50 Case/Control TEST points. **(A)** Examples of networks for two Controls (samples with *R* = 0.01 and *R* = 0.25) and two Cases (samples with *R* = 0.75 and *R* = 0.99). The sizes of the vertices are equal to their strengths; the colours of the vertices indicate the type of feature in the original data (white is the colour of the parameter of the original sphere, grey is the noise variable); the thickness of the edges corresponds to the edge weights [that is, the probability of a sample belonging to a class of cases on the plane of two features (vertices)]; the colours of the edges additionally emphasize their weight *w* (grey: if 0 < *w* ≤ 0.25, yellow: if 0.25 < *w* ≤ 0.5, orange: if 0.5 < *w* ≤ 0.75 and red: if 0.75 < *w* ≤ 1. As can be seen from these examples, the networks of Controls and Cases visually represent different topological objects (the strength of the vertices of the Cases is noticeably greater than the strength of the vertices of the Controls). Moreover, in such networks, the vertices “defining” the difference between the two classes are immediately distinguished (that is, the vertices of the sphere parameters (labeled *X*) are predominantly larger than the vertices of noise variables (labeled *N*) for Cases and less for Controls). **(B)** Distributions of nodes strengths of samples networks, demonstrating the similarity of distributions inside the Control group and inside the Case group and the difference in distributions between groups. **(C)** Boxplots of the nodes strengths in the groups of sphere variables and noise variables, demonstrating that the strengths of the most significant vertices differ significantly from the noise ones within the network of each sample (that is, the greater the weight of the vertex in the Cases and the smaller it in the Controls, the greater the role of this parameter for the whole system). Panels **(D)** and **(E)** show the distribution of the features in the dataset. As can be seen, the distributions of Cases and Controls on the original data do not show clear difference, and the boxplots demonstrate that the noise and sphere variables, on average, do not differ for each sample. Thus, we demonstrate that the transition from original data to networks simultaneously solves two problems: it defines the space in which the Cases and Controls are topologically distinguishable and highlights the group of parameters that are most important and significant for the entire system.

**FIGURE 3:**
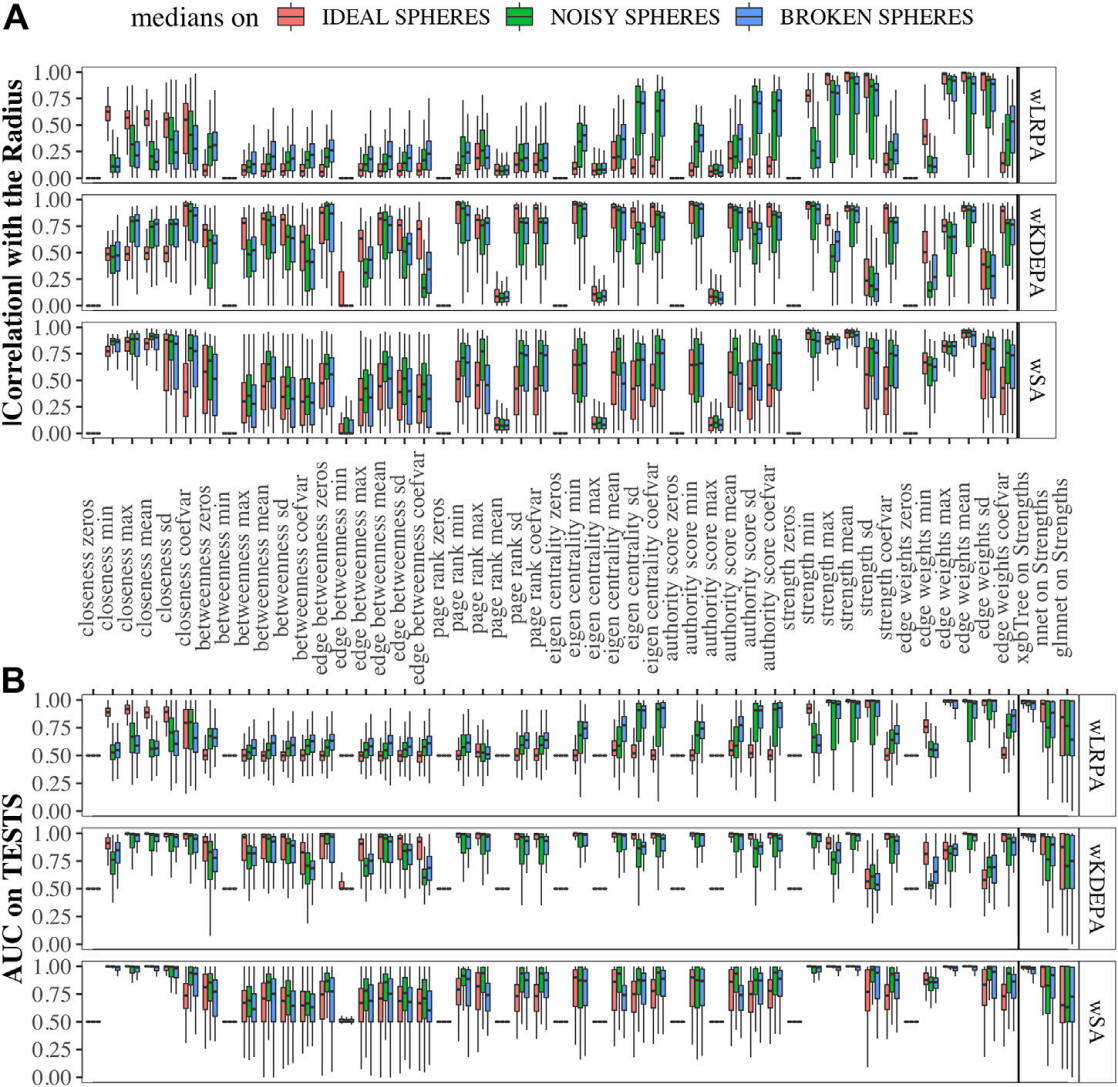
Correlation of network characteristics with sample radii and performance (AUC) of class separation for each network characteristic **(A)** Absolute values of Pearson’s correlation coefficient of 48 descriptive statistics of network characteristics with synthetic-samples’ radii (only TEST subsets were considered). **(B)** AUCs from glm models calculted from network characteristics for TEST folds, and on ML models using strength characteristics (to the right of the verticle line). Models were calcualted for “ideal spheres” (red), “noisy spheres” (green) and “broken spheres” (blue).

Data sets were generated varying the number of dimensions, cases and controls, such as for each sphere-model, 288 different datasets are generated (see Section 2.1). In each dataset and for each network-characteristic, we calculated the absolute value of Pearson’s correlation coefficient between it and the radius of the samples (Figure 3A). We specify here following conclusions:

• For wLRPA, the topological characteristic that has the greatest degree of correlation is the maximum weights of the edges. This is despite the fact that in each plane, the control distribution is poorly described by linear regression (Figure 1B). When considering the maximum all of edge weights, there is typically always a pair of features for a “case” that is a long way from the regression fit, whereas “controls” are always close to the line. Therefore, considering the most extreme point for every case/control, rather than an average is a good correlator in these synthetic data sets.
• For wKDEPA, a fairly large number of characteristics show a good correlation, in particular, the mean of the edge-weights and the mean of the strengths of the vertices. An interesting finding is that the correlation for the models of “noisy spheres” is very inferior to the other two models (comparing the highest correlation between each topological feature), which most likely indicates overfitting of the wKDEPA on noise variables.
• wSA networks demonstrate the best results out of all three networks. Characteristics such as mean of edge weights, mean of vertex strengths, and mean of closeness show very high correlation across all datasets and any model of Spheres (see examples of such networks on Figure 2A) and their strengths distribution on Figures 2B,C). From our point of view, this indicates that the construction of wSA is more advantageous and the established rule for the weight of edges (through the probability of belonging to a class of cases) is reasonable as a measure of the distance from the *center of normality.* It is interesting that for some topological characteristics, such as PageRank, the correlation for the ideal sphere is worse. The reason for this may be that the high-quality prediction generated by the SVMs for each feature minimise the variation in edge weights, and this reduces the capacity of some topologies to change (such as PageRank).

As expected, for those characteristics with a high correlation with the radii, a high quality of class separation was obtained. For each dataset, for each topological characteristic, we trained a glm model on the TRAIN folds and applied it to the TEST folds. With the obtained probability, we calculated the AUCs (area under the receiver operator characteristic curve) and combined them into boxplots for each sphere model (Figure 3B). In addition, we added the results of constructing ML models on the strengths of vertices (since each sample within the network can be represented not by the vector of original features, but by the vector of their strengths in the networks (see examples of wSA networks in Figure 2A and their strengths distribution in Figures 2B,C) which demonstrate an advantage over the distributions of raw features) and found that the results of *xgbTree* for wLRPA and wSA networks are comparable, with the best results on individual characteristics. For wKDEPA, they greatly exceed the results of individual characteristics (and become comparable with the results of wLRPA and wSA). Despite the fact that wKDEPA and wLRPA approaches work a little worse than the wSA, the transformation of the original features to the vertex strengths is an equal substitution, regardless of the choice of the parenclitic approach.

#### 3.1.2 Comparison with Other ML Models on Synthetic Data

**FIGURE 4:**
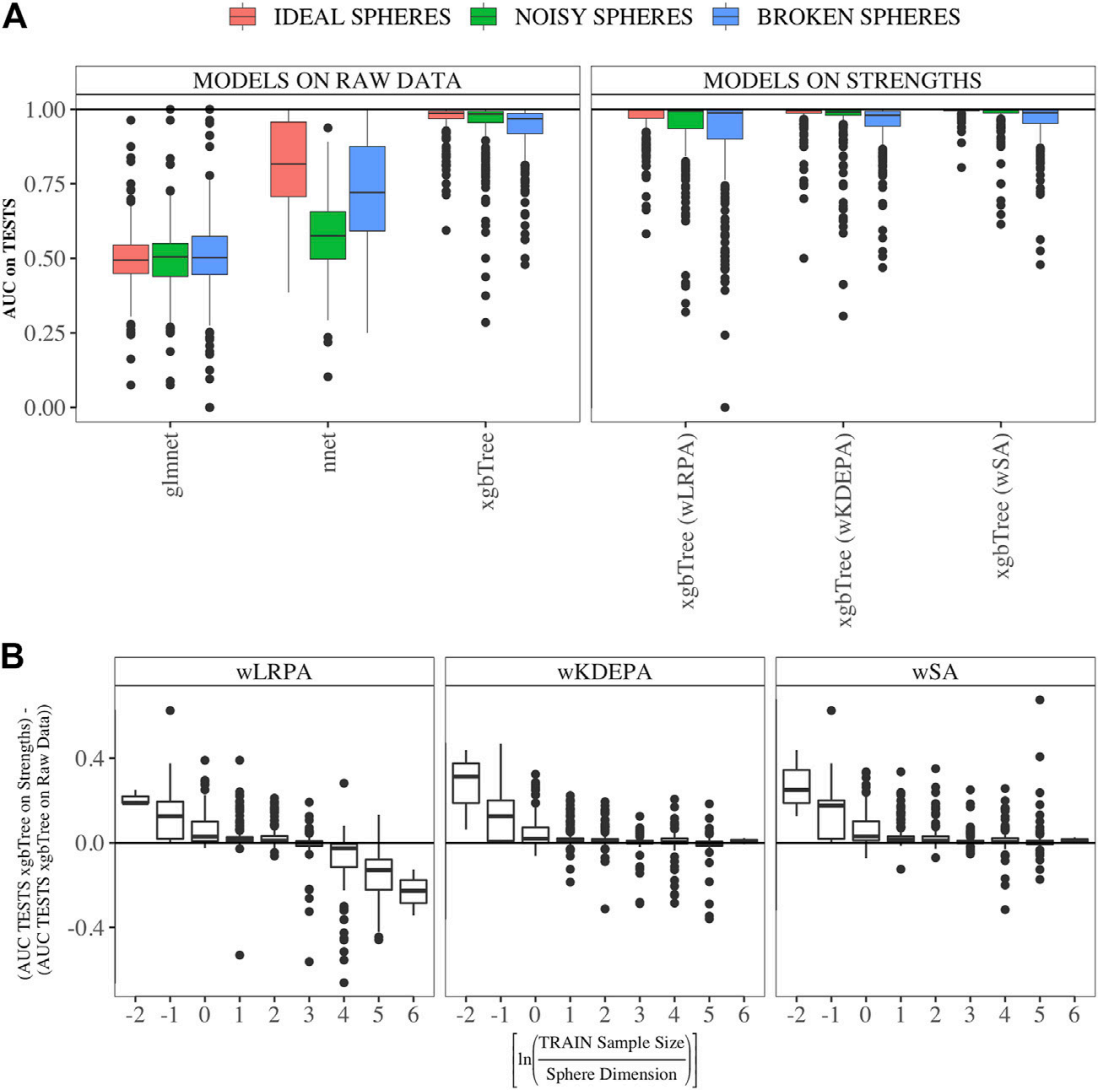
Comparison of the results of parenclitic approaches (the xgbTree model trained on the strengths of vertices) with ML methods (glmnet, nnet, xgbTree) on synthetic data **(A)** Parenclitic analysis demonstrated greater performance (for all three network approaches) than glmnet and nnet on the original data. wSA and wKDEPA approaches demonstrate a slight improvement over xgbTree on the original data. **(B)** The difference of AUC-xgbTree-on-vertex-strengths and AUC-(xgbTree/nnet/glmnet)-on-raw data versus $\left[ln\left(\frac{TRAIN\;Sample\;Size}{Dimension}\right)\right]$ , where [ ] denotes the standard rounding function. Parenclitic approaches on average demonstrate superiority to other ML methods in situations where sample size is small raltive to the number of features.

We compared the quality of parenclitic approaches (for simplicity, we compared only the results of xgbTree on the vertex strengths, since, as can be see from Figure 3B, the nnet and glmnet models worked worse) with 3 ML models on the syntethic datasets (Figure 4). The ML model that produced the most accurate classification of this data was xgbTree (Figure 4A). The results of all the parenclitic approaches produced exceptionally good classification, however, wKDEPA and wSA had extremely positively skewed distributions, meaning that these more frequently gave better classification than other approaches. When considering the impact of sample size, it can be seen that parenclitic models outperform glmnet, nnet and xgbTree when the sample size is small relatively to the spheres dimension (Figure 4B), which most likely indicates that parenclitic approaches are less prone to overfitting. This property itself (apart from other advantages of parenclitic approaches) can be valuable in biological and medical problems utilising omics data, where there can be a huge number of features with comparatively few patients. This is exemplified in Demichev et al. (2021), where the use of wSA was more effective than other ML methods, most likely because the sample size was small relative to the large number of features. It was also seen that for wLRPA, the quality of discrimination (compared to other ML techniques) decreased once the number of samples highly exceeded the number of features, whereas there was no such effect for wKDEPA and wSA.

### 3.2 Comparison of Approaches with Real Data

#### 3.2.1 Parenclitic

**FIGURE 5:**
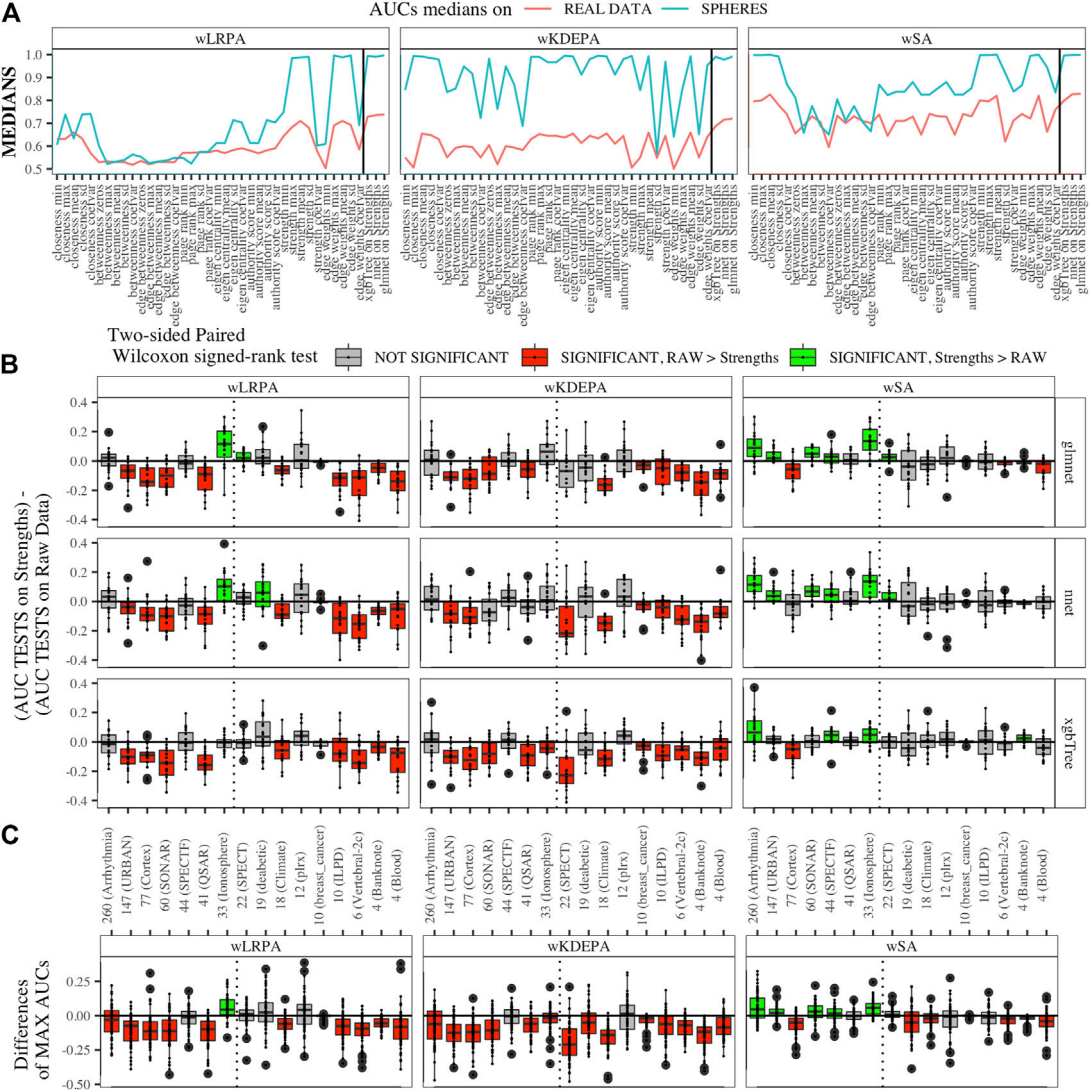
Applying parenclitic approaches to real data **(A)** Medians of AUCs obtained by parenclitic approaches on synthetic data and on real data. On **(B)** and **(C)** we order the datasets by the feature dimension (we display the dimensions on the *x*-axis along with the names of the datasets), we draw a dashed line detaching the datasets to the left that satisfy our expectations to get an advantage of parenclic approaches to them (that is, for which the $\left[ln\left(\frac{40}{Dimension}\right)\right]\leq 0$ , is correct, as 40 is the TRAIN fold size for all subsets). **(B)** The difference between the performances of ML models built on vertex strengths and ML models built on raw data. The effect found on the synthetic data was not confirmed for the wLRPA and wKDEPA approaches, but it was mainly obtained for the wSA: for 6/7 datasets the performances are either comparable or give a gain in the wSA. **(C)** The difference between MAX AUC among 3 models on raw data and among 3 models on strengths data. For wSA approach, on 5/7 datasets to the left of the dotted line, the best ML model on vertex strengths shows a significant advantage than the best ML model on raw data; on 1/7 datasets the results were comparable, and on 1/7 datasets the result was worse.

We generated models for 16 real data sets and calculated the median AUCs for each of the networks characteristics (Figure 5A). For real data, wSA networks performed much better than wLRPA and wKDEPA. Moreover, it is interesting that for wSA networks, the performance of each topological characteristics’ AUC mirrored the AUCs from the synthetic data with closeness mean, strengths mean and weights mean performing strongly in all instances. This most likely indicates that the characteristics of the wSA networks have some conservatism (in terms of the quality of separation), regardless of the data type. We would also like to note the repetition of the effect found on the spheres models. For all three parenclitic approaches, the models built with the vertex strengths give the best performance. Most likely, the fact that the medians of AUC for wKDEPA and wLRPA are low for real data, but the quality of models based on the node strengths is high (although lower than for wSA), indicating that, despite the described shortcomings, such networks are correctly distinguishing classes, but the effects on characteristics are not conservative (that is, the quality of the separation for each characteristic depends on the data type).

#### 3.2.2 Comparison with Others ML Models

Comparison of ML models built on the strengths of vertices and on raw data was carried out for each network approach separately.

Inside each real dataset, for each of the 20 subsets we calculated the quality of each model (AUC on TEST fold) on the raw data and its quality on the vertex strengths. For each main dataset, we get two vectors of length 20 with AUC on TEST for vertices and AUC on TEST for raw data.

First, we calculated the difference between these two vectors for each main dataset and presented them as boxplots in Figure 5B. We also computed a two-sided paired Wilcoxon signed-rank test for each pair of such vectors. If *p*-value ≥ 0.05 (i.e. insignificant), we use gray color for the corresponding box; if *p*-value < 0.05 (i.e. significant), we additionally check which median results (on raw or on strengths data) more, and use red color if more is raw results, and use green color if more is strengths results.

Datasets are sorted in descending order of number its of features. As it was established on synthetic data (Figure 4B) that parenclitic approaches have the greatest advantage on data where the logarithm of the ratio of the sample size in the TRAIN fold to the feature size is no more than 0 (rounded to the nearest real number). Considering that all subsets have sample size of TRAIN folds is 40 samples, among them, datasets with a dimension greater than 33 have this property (indicated by vertical line in Figure 5B).

As shown in Figure 5B, the wLRPA gave only one advantageous result (green box) for the glmnet and nnet models on datasets with a ≥33 dimensions; moreover, in 4 out of 7 of these cases this approach turned out to be worse than the model based on non-parenclitic data. A similar situation was seen for the wKDEPA approach (where not a single winning situation was found on any dataset). On the other hand, for the wSA approach, the advantage of using the wSA approach is clearly seen in sample sets with a large proportion of dimensions compared with other observations (the only exception is the Cortex dataset, in all other cases the wSA works better or comparable than the models on non-parenclitic data).

Additionally, for each individual subset, we calculated the best result (maximum AUC) for 3 models on non-parenclitic data and for 3 models on strengths data and examined the difference in such values (Figure 5C). For the wSA approach, on 5 out of 7 datasets with dimensions ≥33, the best ML model on vertex strengths (that is, using the parenclitic approach) shows a significant advantage than the best ML model on the raw data; on 1 out of 7 datasets (QSAR) the results were comparable, and on 1 out of 7 datasets (Cortex) the result was worse.

## 4 Discussion

The results presented in this work show that the quality of the wSA is comparable to or better than other ML models, if we consider them as classifiers, and better than other parenclitic approaches. At the same time, this approach has several advantages. The transformation of the initial data features into individual networks for each sample facilitates the visualization of the relationship between features, identifying the most significant relationships and the most significant features (e.g. Figure 2A). This approach allows one to build generalizing networks and get an idea of the whole system of interdependencies between features. New rules can be developed to simplify networks (for example, by removing all edges that are not informative in terms of class separation), or highlight hubs, triangles, and use other advanced network analysis procedures. Moreover, as we were able to show with synthetic data and then confirm on real data, the quality of the wSA as a classifier is higher for those datasets where the sample size is small in comparison with the features size. Using this advantage we have recently applied this approach to the analysis of proteomics data from a large cohort of CoVID-19 patients, in which this is the case (Demichev et al., 2021) (manuscript submitted). In this analysis, we showed that wSA was able to produce accurate classifications, where other ML algorithms were not on the same data.

The disadvantages of parenclitic approaches include high computation times (since the construction of models occurs at each pair of features), and certain data structures for which this method will not work. For example, a simple “chess” three-dimensional cube (please rotate the example here)—where the points of cases and controls are grouped similarly to black and white squares on a chessboard. At the same time, despite the fact that the spatial separation of classes obviously exists, in all of the three projections (any two of the three parameters), parenclitic approaches will be not able to detect a qualitative separation (since the points of cases and controls will be mixed on the two-dimensional plane). Despite the fact that wLMPA and wKDEPA approaches did not work much better for classification problems, they are particularly useful in situations where the case group is not known, as they only use the control group for building models, and therefore highlight groups that deviate greatly from controls. This in contrast to wSA models which require pre-defined case/control groups to construct parenclitic networks.

The development of such approaches for application on longitudinal data is of particular interest. As we have demonstrated with the sphere models, some of the characteristics of the parenclitic networks are highly correlated with radii (which in these models is a measure of the deviation from normality). This may mean that the characteristics of parenclitic networks can themselves be the indicators of the development of the disease and can be traced over time to diagnose the onset of the disease. It has been established that the use of longitudinal models (i.e. models that use all historical data for a subject to predict a future or current state) reduces the time to diagnosis for ovarian cancer (Blyuss et al., 2018; Whitwell et al., 2020). Topologies of parenclitic networks (and combinations of topologies) can naturally be incorporated into longitudinal algorithms. Given the power of these approaches individually, the development of their combined use is now a research priority.

To summarise our approach as an instruction for multi-disciplinary researchers:

• For specialists in the field of medicine and biology, using the wSA approach
  ∗ As a classifier, in situations where the number of samples is small in comparison with the dimension of analytes [when there are few patients, but there are many measurements of their states, see, for example, (Demichev et al., 2021) (manuscript submitted)];
  ∗ As a high-quality and simple data visualization, when a visual representation of the state of the system features of an individual in the aggregate is required (we assume that such a representation in the form of networks can give a new understanding of the relationship of features, both among the entire set of subjects, and with an indication of some of their individual properties subjects, as shown in Figure 2A);
  ∗ In situations where it is required to determine the intermediate state of the points during the transition, for example, from a healthy to severely ill state. As we have shown (through the radii on the artificial data, see Figure 3A), parenclitic approaches reflect the spatial state on a one-dimensional scale;
  ∗ When it is required to interpret the transition between two states with respect to some kind of continuous effect (for example, in the work Krivonosov et al. (2020) we showed how the third groups of samples according to the characteristics of networks demonstrate age tendencies between the features selected in binary networks between case and control groups);
• For specialists in the field of machine learning and network approaches, we recommend using the wSA approach, as an interesting method to get new representations of the data. In particular,
  ∗ One can play with a choice of a model to split classes for each pair of features (currently we used everywhere a radial SVM, but we assume that each edge may have its own model, the main thing is that the edge weight is set as the probability of belonging to the same class); or with different classes of models, used on the vertices strengths (or other vectors of characteristics) of the networks.
  ∗ It is possible to have artificial data for which the synolytic approach in this form is not applicable (for example, a “chess” three-dimensional cube described above). We believe that the Cortex data, on which the wSA approach has not received an advantage, were of similar type. However, it would be interesting to extend this approach to consider not only pairs, but also triplets and quadruples of features with the correct collection of the results into an edge between two features (to continue to obtain a structure on the graph).

Finally, our approach, if combined with artificial neural networks, may contribute to the development of explainable artificial intelligence, because network visualisation assists the understanding in each step of data processing.

## Data Availability

The original contributions presented in the study are included in the article/Supplementary Material, further inquiries can be directed to the corresponding author.
