# Supplementary material for: Parenclitic and Synolytic Networks Revisited
Source: Front Genet. 2021 Oct 20;12:733783. doi: 10.3389/fgene.2021.733783 (PMC8564045; doi:10.3389/fgene.2021.733783)
Supplement: Supplementary file 1 [file Presentation1.PDF]

Supplementary Files

REAL DATA

LINK: https://liveuclac-my.sharepoint.com/:f:/g/personal/rmjotzn_ucl_ac_uk/Eiendh0eZRNIsvQaEQ4TL_cBlP7Mrow96n2UfXOxS9QCTw?e=WMV7ro

Data of real data datasets (which was downloaded from https://archive.ics.uci.edu and pre-processed).
Folder contains directories

- **ORIGINAL_FULL_DATA**:
  each file
  (called as *Name_Of_Main_Dataset*.csv, where *Name_Of_Main_Dataset* = **Arrhythmia/Banknote/Blood/breast_cancer/Climate/Cortex/diabetic/ILPD/Ionosphere/plrx/QSAR/SONAR/SPECT/SPECTF/URBAN/Vertebral-2c**)
  contains columns of parameters and additional labels:
  - score – label of classes (case or control);
  - id – indicator of sample;
- **ORIGINAL _SUBSET_DATA**:

each file
(called as *Name_Of_Main_Dataset_20_ITER*.csv, where *ITER* = Iteration of sub-selected sample (number from 1 to 20)
contains columns of parameters and additional labels:

- score – label of classes (case or control);
- label – label of fols (TRAIN or TEST)
- id – indicator of sample;

- **NETWORKS**:

each file
(called as (**wLRPA/wKDEPA/wSA**)_*Name_Of_Main_Dataset_20_ITER*_networks.csv)
contains corresponding **wLRPA/wKDEPA/wSA** network for dataset:
first 2 columns (p1,p2) indicates pair of parameters (vertices) forming an edge;
other columns (from 3 and up) indicate vectors of edges weights for each sample id from ORIGINAL _DATA table.

- **CHARACTERISTICS**:

each file
(called as (**wLRPA/wKDEPA/wSA**)_ *Name_Of_Main_Dataset_20_ITER*_chars.csv)
contains 48 network characteristics and additional labels (score, label, id corresponding to original data)

and file of results ***all_results_for_realdata_datasets.csv*** where columns are:

Name_Of_Main_Dataset – name of corresponding dataset
Number_Of_Features_in_Main_Dataset – number of features in main dataset
Name_Of_Subset – name of corresponding subset (*Name_Of_Main_Dataset_20_ITER*.csv, where *ITER* = Iteration of sub-selected sample (number from 1 to 20))
Number_Of_Features_in_Subset - number of features in subset (this number can be slightly different from *Number_Of_Features_in_Main_*Dataset if on selected sample some parameters have sd = 0)
Type_Of_Result – “MODELS ON ROW DATA” or “GLM ON 1 CHARACTERISTIC” or “MODELS ON STRENGTHS”
model – IF *Type_Of_Result* is “GLM ON 1 CHARACTERISTIC”, then *model* is one of 48 network characteristics,
ELSE *model* is one of the ML model “glmnet” or “nnet” or “xgbTree”
Type_of_Network – “wLRPA” or “wKDEPA” or “wSA”
AUC_train – result (AUC) on TRAIN fold
AUC_test – result (AUC) on TEST fold
TRAIN_NUMBER_CASES – number of cases in TRAIN fold *(always 20)*
TRAIN_NUMBER_CONTROLS – number of controls in TRAIN fold *(always 20)*
TEST_NUMBER_CASES - number of cases in TEST fold *(always 20)*
TEST_NUMBER_CONTROLS - number of controls in TEST fold *(always 20)*

SPHERES

LINK: https://liveuclac-my.sharepoint.com/:f:/g/personal/rmjotzn_ucl_ac_uk/En6FI_LAxypKirGHTRkkA7AB4RkRcXvARGUEgOcloWy3Iw?e=80nFqw

Spheres datasets (which were generated). Folder contains directories

- **ORIGINAL_GENERATED_DATA**:

each file

(called as (ideal/noisy/broken)_*Name_Of_Data*.*csv*, where *Name_Of_Data* = "sphere_*N_NCsTr_NCnTr_NCsTs_NCnTs",* where *N* – dimensionality of the corresponding ideal sphere, *NCsTr* - number of cases in TRAIN fold, *NCnTr* - number of controls in TRAIN fold, *NCsTs* - number of cases in TEST fold, *NCnTs* - number of controls in TEST fold)

contains columns of parameters and additional labels:

- score – label of classes (case or control);
- label – label of fols (TRAIN or TEST)
- id – indicator of sample

- **NETWORKS**:

each file

(called as (**wLRPA/wKDEPA/wSA**)_(ideal/noisy/broken)_*Name_Of_Data*_networks.csv)

contains corresponding **wLRPA/wKDEPA/wSA** network for dataset:

first 2 column (p1,p2) indicates pair of parameters (vertices) forming an edge;

other columns (from 3 and up) indicate vectors of edges weights for each sample id from ORIGINAL _DATA table.

- **CHARACTERISTICS**:

each file

(called as (**wLRPA/wKDEPA/wSA**)_(ideal/noisy/broken)_*Name_Of_Data*_chars.csv)

contains 48 network characteristics and additional labels (score, label, id corresponding to original data)

and file of results ***all_results_for_sphere_datasets.csv*** where columns are:

Name_Of_Data – name of corresponding dataset

Type_Of_Sphere – type of sphere: "IDEAL SPHERES" or "NOISY SPHERES" or "BROKEN" spheres

Type_Of_Result – "MODELS ON ROW DATA" or "GLM ON 1 CHARACTERISTIC" or "MODELS ON STRENGTHS"

model – IF *Type_Of_Result* is "GLM ON 1 CHARACTERISTIC", then *model* is one of 48 network characteristics, ELSE *model* is one of the ML model "glmnet" or "nnet" or "xgbTree"

Type_of_Network – "wLRPA" or "wKDEPA" or "wSA"

AUC_train – result (AUC) on TRAIN fold

AUC_test – result (AUC) on TEST fold

S_DIMENSION – dimension of corresponding ideal sphere

TRAIN_NUMBER_CASES – number of cases in TRAIN fold

TRAIN_NUMBER_CONTROLS – number of controls in TRAIN fold

TEST_NUMBER_CASES - number of cases in TEST fold

TEST_NUMBER_CONTROLS - number of controls in TEST fold
